# Prehospital Testing and Surveillance for SARS-CoV-2: A Special Report from the Sacramento (California USA) Mobile Integrated Health Unit

**DOI:** 10.1017/S1049023X22000292

**Published:** 2022-02-11

**Authors:** Angela F. Jarman, James S. Ford, Matthew J. Maynard, Zena L. Simmons, Kevin E. Mackey, Bryn E. Mumma, John S. Rose

**Affiliations:** 1. Department of Emergency Medicine, University of California-Davis, Sacramento, California USA; 2. United States Air Force; 3. School of Medicine, University of California-Davis, Sacramento, California USA; 4. Department of Emergency Medicine, Kaiser Permanente, Sacramento, California USA; 5. Sacramento Regional Fire and Emergency Communication Center, Sacramento, California USA

**Keywords:** congregate living, COVID-19, disease surveillance, health equity, mobile care delivery, SARS-CoV-2, ALF, assisted living facilities, BCF, board and care facility, CDC, Centers for Disease Control and Prevention, CMS, Centers for Medicaid and Medicare Services, COVID-19, coronavirus disease 2019, EMS, Emergency Medicine Services, MIH, Mobile Integrated Health Unit, MIH-CP, Mobile-Integrated Healthcare Community Paramedicine, PSY, inpatient psychiatric facilities, SARS-CoV-2, Severe Acute Respiratory Syndrome Coronavirus 2, SCDPH, Sacramento County Department of Public Health, SNF, skilled nursing facilities

## Abstract

**Introduction:**

Coronavirus disease 2019 (COVID-19), caused by Severe Acute Respiratory Syndrome Coronavirus 2 (SARS-CoV-2), has killed nearly 800,000 Americans since early 2020. The disease has disproportionately affected older Americans, men, persons of color, and those living in congregate living facilities. Sacramento County (California USA) has used a novel Mobile Integrated Health Unit (MIH) to test hundreds of patients who dwell in congregate living facilities, including skilled nursing facilities (SNF), residential care facilities (ie, assisted living facilities [ALF] and board and care facilities [BCF]), and inpatient psychiatric facilities (PSY), for SARS-CoV-2.

**Methods:**

The MIH was authorized and rapidly created at the beginning of the COVID-19 pandemic as a joint venture between the Sacramento County Department of Public Health (SCDPH) and several fire-based Emergency Medical Services (EMS) agencies within the county to perform SARS-CoV-2 testing and surveillance in a prehospital setting at a number of congregate living facilities. All adult patients (≥18 years) who were tested for SARS-CoV-2 infection by the MIH from March 31, 2020 through April 30, 2020 and lived in congregate living facilities were included in this retrospective descriptive cohort. Demographic and laboratory data were collected to describe the cohort of patients tested by the MIH.

**Results:**

During the study period, the MIH tested a total of 323 patients from 15 facilities in Sacramento County. The median age of patients tested was 66 years and the majority were female (72%). Overall, 72 patients (22%) tested positive for SARS-CoV-2 in congregate living settings, a higher rate of positivity than was measured across the county during the same time period.

**Conclusion:**

The MIH was a novel method of epidemic surveillance that succeeded in delivering effective and efficient testing to patients who reside in congregate living facilities and was able to accurately identify pockets of infection within otherwise low prevalence areas. Cooperative prehospital models are an effective model to deliver out-of-hospital testing and disease surveillance that may serve as a blueprint for community-based care delivery for a number of disease states and future epidemics or pandemics.

## Introduction

Coronavirus disease 2019 (COVID-19), which is caused by Severe Acute Respiratory Syndrome Coronavirus 2 (SARS-CoV-2), was declared a pandemic by the World Health Organization (WHO; Geneva, Switzerland) in March 2020.^1^ To date, over 260 million cases of COVID-19 and over 5.2 million associated deaths have been confirmed world-wide.^2^ The burden of disease has been particularly high in the United States, causing detrimental effects on the health care system and economy and leaving many Americans unemployed, disabled, or deceased.^3,4^ In California alone, 4.8M patients have been infected and over 73,000 have died.^5,6^ Sacramento County (population 1.5M), which includes the capitol city of Sacramento, California, has seen over 2,350 deaths from its 163,000 cases.^7^

Epidemiologic data on COVID-19 infections in multiple countries suggest increasing mortality associated with older age, comorbid conditions (particularly chronic heart and lung disease and diabetes), and male sex.^8–10^ Those living in congregate living facilities such as correctional facilities, skilled nursing facilities (SNF), and inpatient psychiatric facilities (PSY) have suffered a disproportionate burden of disease due to a combination of individual and population level influences.^8,9,11–14^ In one study of an infection cluster in a SNF, asymptomatic infection occurred in nearly 40% of patients and 30-day mortality was 29%.^15^ Congregate living facilities face challenges in conducting whole-facility surveillance through standard means, highlighting the need for novel testing strategies that can meet the unique circumstances of these facilities.

In 1996, the US National Highway Traffic Safety Administration (NHTSA; Washington, DC USA), which serves as an important regulatory body for US-based Emergency Medicine Services (EMS), delivered an “Agenda for the Future” which outlined a vision in which EMS would: “…have the ability to identify and modify illness and injury risks, provide acute illness and injury care and follow-up, and contribute to treatment of chronic conditions and community health monitoring.”^16^ In the ensuing decades, this publication spurred the creation of Mobile-Integrated Healthcare Community Paramedicine (MIH-CP) as a model for low-cost, community-focused health care delivery by EMS practitioners.^17^ A systematic review of such MIH-CP programs found they were associated with decreased emergency department visits, lower hospital admission rates, decreased health expenditures, improved disease-specific metrics for chronic conditions such as diabetes and hypertension, and improved patient satisfaction.^18^

In this study, a novel application of MIH-CP is described as a COVID-19 pandemic surveillance model in congregate living facilities in Sacramento County. The aim was to evaluate the effectiveness of this model and to describe the initial cohort of patients that it served.

## Methods

At the outset of the COVID-19 pandemic in Northern California, a Mobile Integrated Health Unit (MIH) was authorized and created to address various medical needs within Sacramento County. The Sacramento County MIH project was a joint venture between the Sacramento County Department of Public Health (SCDPH; Sacramento, California USA) and the fire-based EMS assets of the Sacramento Metropolitan Fire District (SMFD; Mather, California USA), City of Sacramento Fire Department (SFD; Sacramento, California USA), and Cosumnes Fire Department (CSD; Elk Grove, California USA). The goal of the MIH was to unburden the health care system and decrease utilization of resources by decreasing unnecessary hospital and clinic visits during a period of significant strain caused by the COVID-19 pandemic. The MIH program provided an opportunity to perform SARS-CoV-2 testing and surveillance in a large number of congregate living facilities; these included SNFs, assisted living facilities (ALFs), board and care facilities (BCFs), and PSY. The SCDPH identified daily testing sites based on current SARS-CoV-2 infection surveillance assessment protocols. This process used county-level case tracking data to identify high-risk patients and high-yield settings for potential outbreaks. Specifically, priorities were generated based on those with known exposures and COVID-19 positive hospital discharges in congregate living settings. Each day, SCDPH assigned sites to which the MIH responded for testing and treatment.

All SARS-CoV-2 swabs, which were collected by a licensed health professional (physician or advanced practice provider), were processed by the SCDPH. During the period of analysis, the Centers for Disease Control and Prevention (CDC; Atlanta, Georgia USA) 2019-novel coronavirus reverse transcription polymerase chain reaction (RT-PCR) test assay was utilized; detailed test performance characteristics have been previously published.^19^

### Study Design

This was a retrospective cohort study of deidentified data collected prospectively by the MIH in Sacramento County. Given that the deidentified data were part of a quality improvement project, it was deemed exempt by the University of California, Davis IRB (Davis, California USA). All adult patients (≥18 years) who were tested for SARS-CoV-2 infection by the MIH and lived in licensed congregate living facilities were included; no exclusion criteria were applied. Data were collected from March 31, 2020 through April 30, 2020, which represents a period of high SARS-CoV-2 infection prevalence following the first surge in Sacramento County.^20^ Data collected included date of testing, residence type, and SARS-CoV-2 test result; patients self-reported demographic data, including age, sex, and race. While most patients were verbal and had proficient English literacy, in cases where disability or limited literacy prevented patients from completing a written demographic questionnaire, facility staff may have provided assistance. Residence type was classified based on Centers for Medicaid and Medicare Services (CMS; Baltimore, Maryland USA) designation for SNF and PSY. All residential care facilities including ALF and BCF were designated and licensed by the state of California.^20^ Of note, there are a total of 930 long-term care facilities that are licensed in the county, which includes all non-SNF facilities.^22^

### Data Analysis

Descriptive statistics of the data were performed. Categorical variables were reported as percentages and proportions. Continuous variables were presented as medians and interquartile ranges (IQR). Due to relatively small sample sizes, the Fischer’s Exact test was used to compare categorical variables. Due to the non-parametric distribution of certain variables, the Mann-Whitney U test was used to compare continuous variables. Age was analyzed as a continuous variable. Biological sex was modeled as binary (male/female) and race was also modeled as binary (White/non-White) given the small sample sizes of non-White racial/ethnic groups. All data were analyzed using Stata 15 (StataCorp; College Station, Texas USA).^23^

## Results

A total of 323 patients from 15 facilities in Sacramento County were tested for SARS-CoV-2 and included in the analysis. Patients were tested from a total of two of the six PSY, six of the 37 SNF, six ALF, and one BCF in Sacramento County. The median age of patients tested was 66 years (IQR 46-85) and the majority were female (72%; 232/323). Race data were unavailable in two patients. The predominant race in this cohort was White (63%; 203/323), followed by Asian (15%; 47/323), Hispanic/Latinx (12%; 40/323), and Black (8%; 26/323). Most patients lived in SNF (59%; 192/323). Patient characteristics are available in Table 1.

**Table 1.** Patient Characteristics and SARS-CoV-2 Positivity

	All Patients (n = 323)	SARS-CoV-2+ Patients (n = 72)
**Median Age (years)**	66 (46-85)	82 (53-90)
**Female Sex**	72% (232/323)	69% (50/72)
**Race/Ethnicity**		
White	63% (203/323)	76% (55/72)
Asian	15% (47/323)	7% (5/72)
Hispanic	12% (40/323)	8% (6/72)
Black	8% (26/323)	4% (3/72)
Other/Mixed	2% (5/323)	1% (1/72)
Unknown	<1% (2/323)	3% (2/72)

Note: Categorical variables presented as percentages and proportions. Continuous variables presented as medians and interquartile ranges.

Abbreviation: SARS-CoV-2, Severe Acute Respiratory Syndrome Coronavirus 2.

Of the total patients tested, 72 (22%) tested positive for SARS-CoV-2. There was no difference in positivity rate between males and females, respectively (24% [22/91] versus 22% [50/232]; P = .66). Likewise, race was not a significant predictor of SARS-CoV-2 positivity in this cohort. Older age was associated with SARS-CoV-2 positivity, which was an expected finding based on prior epidemiologic data. The highest rate of positivity by type of congregate living facility was found in patients residing in PSY (50%; 12/24; Table 2). The lowest rate of positivity by type of congregate living facility was found in patients residing in SNF (9%; 18/192). Significant clustering was seen, however, by discrete facility. For both PSY and SNF, one facility accounted for all positive cases within that housing category.

**Table 2.** SARS-CoV-2 Positivity by Facility Type

Facility Type	SARS-CoV-2 Positive
**Skilled Nursing Facilities**	**9% (18/192)**
1	0% (0/21)
2	11% (18/164)
3	0% (0/1)
4	0% (0/1)
5	0% (0/2)
6	0% (0/3)
**Assisted Living Facilities**	**38% (38/100)**
1	54% (14/26)
2	67% (4/6)
3	38% (10/26)
4	15% (5/34)
5	100% (1/1)
6	57% (4/7)
**Board and Care Facility**	**57% (4/7)**
**Psychiatric Treatment Facilities**	**50% (12/24)**
1	52% (12/23)
2	0% (0/1)

Abbreviation: SARS-CoV-2, Severe Acute Respiratory Syndrome Coronavirus 2.

## Discussion

The overall composition of the residential care cohort was consistent with national trends in congregate living facilities. The cohort was 71% female, which is similar to national statistics, and 63% White, demonstrating relative diversity compared to national estimates which are 81% non-Hispanic White.^24^ The rate of SARS-CoV-2 positivity in this cohort (22%) was much higher than the rate of positivity in Sacramento County during the same time period (6.3%-7.8).^20^ As discussed previously, SCDPH guidelines prioritized testing in facilities in which there had been known cases of SARS-CoV-2, which likely contributed to the higher prevalence found in these facilities. This finding is consistent, however, with other reports of high rates of SARS-CoV-2 infection and transmission in congregate living facilities.^11,25^ Numerous outbreaks associated with congregate living facilities, for example SNF and prisons, have demonstrated the challenges in accomplishing “social distancing” in communal living environments.^26^ The particular risk of SNF, given both the congregative living conditions and characteristics of their residents who generally are older and have comorbid medical conditions, have prompted specific guidelines from both the CDC and CMS to mitigate SARS-CoV-2 transmission in this setting.^27,28^ While the majority (59%) of this cohort resided in SNF, this housing type was associated with the lowest risk of SARS-CoV-2 positivity when compared to other types of residential care facilities. The reasons for this are unclear, but may be related the SNF-specific guidance from the CDC and to the higher level of training and certification required in these facilities to meet federally-mandated standards for reimbursement by CMS; this is not required of other residential care facilities such as ALF. While it was not possible to obtain facility-level data on COVID-19 protocols with regard to masking and personal protective equipment (PPE), these data were taken from early in the pandemic in Sacramento County. As such, local COVID-19 mitigation protocols were likely less developed and less stringent at that time. The highest rate of infection was found in one of two included PSY. This population specifically has also been found to be at high risk of transmission of SARS-CoV-2 given the communal nature and design of these facilities and a patient population that is not able to consistently follow masking and social distancing guidelines.^29,30^

These data support that congregate living facilities in general are at a high risk of SARS-CoV-2. It is thus important to raise awareness of SARS-CoV-2 transmission in all types of congregate living facilities that may effectively be thought of as large households, within which it is very challenging to socially distance and prevent transmission of respiratory droplets. This risk affects not just SNF and prisons, which have been well-publicized, but all types of congregate living including smaller residential living facilities like BCF which are not licensed and regulated through CMS.

As has been seen throughout history, pandemics do not affect all populations equally and many under-served communities lack access to high-quality health care.^14^ These challenges have been exacerbated in the COVID-19 pandemic and numerous studies have linked disadvantaged neighborhoods with increased prevalence of COVID-19.^31,32^ Mobile integrated health care is a successful model to deliver community-based care to vulnerable populations, such as those in congregate living, and is deserving of further investigation.

## Limitations

There are several important limitations to this study. Most importantly, it is a retrospective descriptive cohort study. This cohort includes all patients residing in licensed residential facilities tested by the MIH during the study period, however there may be selection bias that was unable to be controlled for retrospectively. Thus, these results are applicable only to patients of similar demographics living in congregate living facilities. In addition, for both SNF and PSY facilities, all positive patients resided in one facility; as such, it is likely that high rates of infection and transmission were related to facility-specific protocols and may not be generalizable to other facilities.

## Conclusion

The MIH program is a novel and feasible way to deliver community-based testing and surveillance during an epidemic to individuals living in congregate living facilities. The Sacramento MIH tested over 300 patients during the month studied, 22% of whom tested positive for SARS-CoV-2. This program was thus able to identify multiple pockets of infection associated with specific facilities within an otherwise low prevalence area. This early identification may allow for more strict quarantine and isolation practices, as well as contact tracing, to limit further spread. This model has shown to be effective and feasible and thus is easily adaptable to other infectious disease outbreaks in the future.
